# Epidemiology and Clinical Symptoms Related to Seasonal Coronavirus Identified in Patients with Acute Respiratory Infections Consulting in Primary Care over Six Influenza Seasons (2014–2020) in France

**DOI:** 10.3390/v12060630

**Published:** 2020-06-10

**Authors:** Shirley Masse, Lisandru Capai, Natacha Villechenaud, Thierry Blanchon, Rémi Charrel, Alessandra Falchi

**Affiliations:** 1 Laboratoire de Virologie, Université de Corse-Inserm, 20250 UR7310 Corte, France; masse_s@univ-corse.fr (S.M.); capai_l@univ-corse.fr (L.C.); villechenaud_n@univ-corse.fr (N.V.); 2 Institut Pierre Louis d’Epidémiologie et de Santé Publique, Sorbonne Université, 75012 Paris, France; thierry.blanchon@iplesp.upmc.fr; 3 Unité des Virus Emergents (UVE: Aix Marseille Université, IRD 190, Inserm 1207, IHU Méditerranée Infection), 13000 Marseille, France; remi.charrel@univ-amu.fr

**Keywords:** human coronavirus, acute respiratory infection, influenza, general practice, respiratory pathogens, influenza season

## Abstract

There is currently debate about human coronavirus (HCoV) seasonality and pathogenicity, as epidemiological data are scarce. Here, we provide epidemiological and clinical features of HCoV patients with acute respiratory infection (ARI) examined in primary care general practice. We also describe HCoV seasonality over six influenza surveillance seasons (week 40 to 15 of each season) from the period 2014/2015 to 2019/2020 in Corsica (France). A sample of patients of all ages presenting for consultation for influenza-like illness (ILI) or ARI was included by physicians of the French *Sentinelles* Network during this period. Nasopharyngeal samples were tested for the presence of 21 respiratory pathogens by real-time RT-PCR. Among the 1389 ILI/ARI patients, 105 were positive for at least one HCoV (7.5%). On an annual basis, HCoVs circulated from week 48 (November) to weeks 14–15 (May) and peaked in week 6 (February). Overall, among the HCoV-positive patients detected in this study, HCoV-OC43 was the most commonly detected virus, followed by HCoV-NL63, HCoV-HKU1, and HCoV-229E. The HCoV detection rates varied significantly with age (*p* = 0.00005), with the age group 0–14 years accounting for 28.6% (*n* = 30) of HCoV-positive patients. Fever and malaise were less frequent in HCoV patients than in influenza patients, while sore throat, dyspnoea, rhinorrhoea, and conjunctivitis were more associated with HCoV positivity. In conclusion, this study demonstrates that HCoV subtypes appear in ARI/ILI patients seen in general practice, with characteristic outbreak patterns primarily in winter. This study also identified symptoms associated with HCoVs in patients with ARI/ILI. Further studies with representative samples should be conducted to provide additional insights into the epidemiology and clinical features of HCoVs.

## 1. Introduction

Coronaviruses (CoVs) are an enveloped, single positive-strand RNA species of viruses belonging to the *Coronaviridae* family, which infect birds and mammals. In animals, CoVs cause respiratory, enteric, cardio-vascular, and neurological disorders [1]. In humans, these viruses result in respiratory and gastro-intestinal symptoms, ranging from cold symptoms to severe diseases [2,3].

CoVs recognized to infect humans belong to the genera *Alphacoronavirus* and *Betacoronavirus* [4]. Seven CoV species are known to cause human infection, of which four HCoVs (namely HCoV 229E, NL63, OC43, and HKU1) are known as non-severe acute respiratory syndrome (SARS)-like CoVs. HCoV-229E and HCoV-NL63 belong to the genus *Alphacoronavirus*. The genus *Betacoronavirus* includes HCoV-HKU1, HCoV-OC43, SARS-CoV-1 [5], the Middle East respiratory syndrome (MERS) coronavirus (MERS-CoV) [6], and the SARS-CoV-2, which is currently associated with a global outbreak [7].

The four non-SARS/MERS species circulate widely in humans and infect individuals of all ages [8,9]. HCoV-229E and HCoV-OC43 were identified in 1967 and were primarily associated with mild upper respiratory tract infections [10,11]. HCoV-NL63 was identified in 2004 from a 7-month-old child suffering from bronchiolitis and conjunctivitis [12] and HCoV-HKU1 was discovered in 2005 in Hong Kong and isolated from patients with pneumonia [13]. In general, the four common circulating HCoVs mostly infect humans during the winter season (December–April) [14], whereas circulation of HCoV-HKU1 has been observed during the spring–summer period [9].

The World Health Organization has highlighted the need to improve epidemiological surveillance and knowledge of the health burden imposed by non-influenza respiratory viruses [15]. HCoVs are generally associated with mild upper respiratory tract infections [16], but severe infections with HCoV-229E, HCoV-NL63, and HCoV-OC43 have been reported [17,18,19].

In the context of the spread of SARS-CoV-2 in the community, a better understanding of the seasonality and clinical features of patients with confirmed HCoVs could be useful for mathematical modelling and clinical diagnosis. There is currently a debate about HCoV seasonality and pathogenicity, as epidemiological data are scarce and mostly from hospitalized populations. Here, we document the epidemiological and clinical features of HCoV patients with acute respiratory infection (ARI) observed in general practice. We also describe HCoV seasonality over six influenza surveillance seasons (2014/2015 to 2019/2020) in Corsica, France.

## 2. Materials and Methods

### 2.1. Clinical Samples

**Figure 1:**
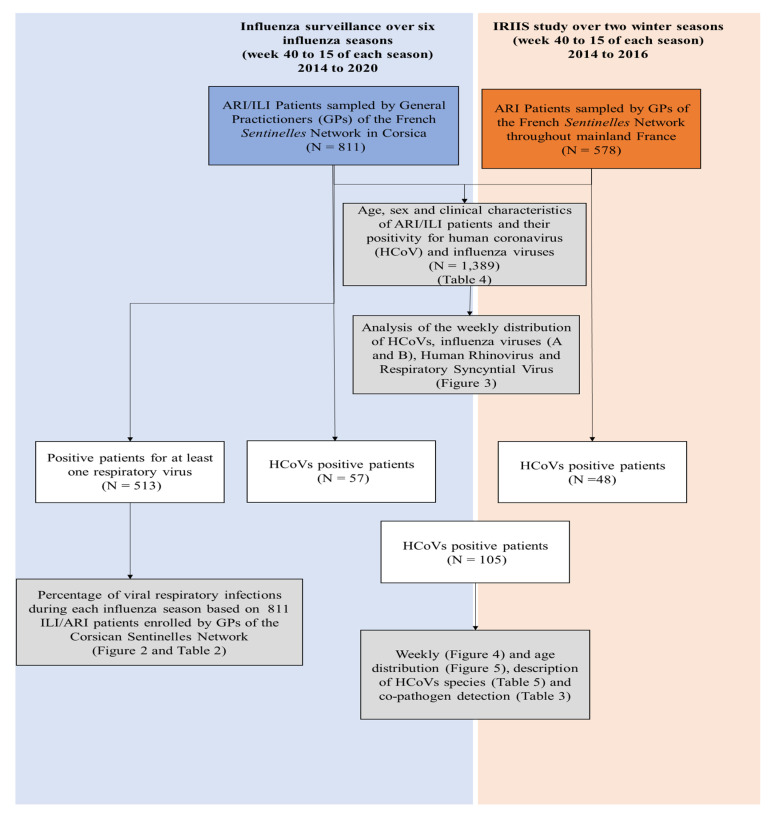
Diagram of the data flow summarizing the patient subsets used for each analysis.

Nasopharyngeal samples were collected: i) as part of the community influenza surveillance conducted in collaboration with the French *Sentinelles* Network from patients seen in general practice, consulting for influenza-like illness (ILI) or ARI (for patients aged >65 years old) during six influenza seasons (week 40 to 15 of each season) from 2014 to 2020 in Corsica, France; and ii) from ARI patients enrolled throughout mainland France by general practitioners (GPs) of the French *Sentinelles* Network, during an epidemiological study of the risk factors for seasonal influenza (IRIIS study; 2014–2016 influenza seasons (week 40 to 15)) [20]. Notably, to ensure that the selection of ILI/ARI patients remained random, each GP was required to include, each week, the first two patients unrelated to one another, consulting within <48 h since symptom onset and consenting to provide a nasopharyngeal specimen. Each patient could be included only once a year (Table 1 and Figure 1). The surveillance uses a specific definition of ILI for patient recruitment: sudden onset of fever >39 °C with myalgia and respiratory signs, diagnosed by the physician. The case definition of ARI was “any person with a sudden onset of symptoms and at least one of the following four systemic symptoms: fever or feverishness, malaise, headache, myalgia, and at least one of the following three respiratory symptoms: cough, sore throat, or shortness of breath.”

### 2.2. Viral Diagnostic Testing

Viral genome was extracted from 200 μL of patient samples using the QIAamp MinElute Virus Spin Kit (Qiagen, France). Routine molecular testing by real-time RT-PCR (rtRT-PCR) using an FTD Respiratory pathogens 21 Kit (Fast Track Diagnostics, Luxembourg) was employed from 2017/2018 onwards to detect: influenza viruses (subtypes A and B: flu A and flu B), human rhinovirus (HRV), human coronaviruses NL63 (HCoV-NL63), 229E (HCoV-229E), OC43 (HCoV-OC43), and HKU1 (HCoV-HKU1), respiratory syncytial virus (RSV), human metapneumovirus (HMPV), human adenovirus (HAdV), human parainfluenza virus (HPIV), and human bocavirus (HBoV). Samples collected in 2014/2015 and 2016/2017 were analysed by ARGENE^®^ Respiratory Range (Marcy l’Etoile, France). This rRT-PCR did not provide details for HCoVs and HAdV, HBoV, and HPIV were not analysed. Thus, samples still available were retrospectively analysed for CoV-229E, CoV-OC43, and CoV-NL63 by FTD.

All nasopharyngeal samples collected by the French *Sentinelles* Network during the 2019/2020 season were analysed for SARS-CoV-2. RdRp-IP1 and RdRp quantitative rtRT-PCR was used for detection of SARS-CoV-2. The RdRp rtRT-PCR corresponds to the Charité protocol [21]. When a sample was positive for RdRp-IP1, quantification of the number of RNA copies was performed according to a scale ranging from 10^3^ to 10^7^ copies per μL [22]. All analyses were conducted by the Laboratory of Virology, University of Corsica.

### 2.3. Seasonality of HCoVs in ILI/ARI Patients in General Practice

The HCoV seasonality in Corsica from 2014/2015 to 2019/2020 was studied using samples collected by GPs of the Corsican *Sentinelles* Network, as constant and homogeneous monitoring of this population of ILI/ARI patients has been conducted throughout six influenza seasons (Table 1 and Figure 1).

To study the weekly number of HCoVs detected among ILI/ARI patients seen in general practice during the six influenza seasons, we gathered all samples collected by GPs for influenza surveillance and for the IRIIS study (Table 1 and Figure 1).

### 2.4. Ethical Considerations

The samples were obtained as part of influenza surveillance. The protocol was conducted in agreement with the Helsinki Declaration. We obtained authorization from the French Data Protection Agency (CNIL#471393). The IRIIS study was approved by the Ethics Committee (CPP SUD MEDITERRANEE V, 01/09/2014; ref. number 14.078).

### 2.5. Statistical Analysis

Differences according to viral infection and epidemiological data (sex, age, clinical symptoms, and risk factors) were analysed and tested using Fisher’s exact test or Chi-square test. Results were considered statistically significant when the *p* value was lower than 0.05. All statistical analyses were performed using R software version 3.6.1 (R Foundation, Vienna, Austria).

## 3. Results

### 3.1. HCoV Prevalence over Six Influenza Seasons among Patients with ILI/ARI Seen in General Practice in Corsica

**Figure 2:**
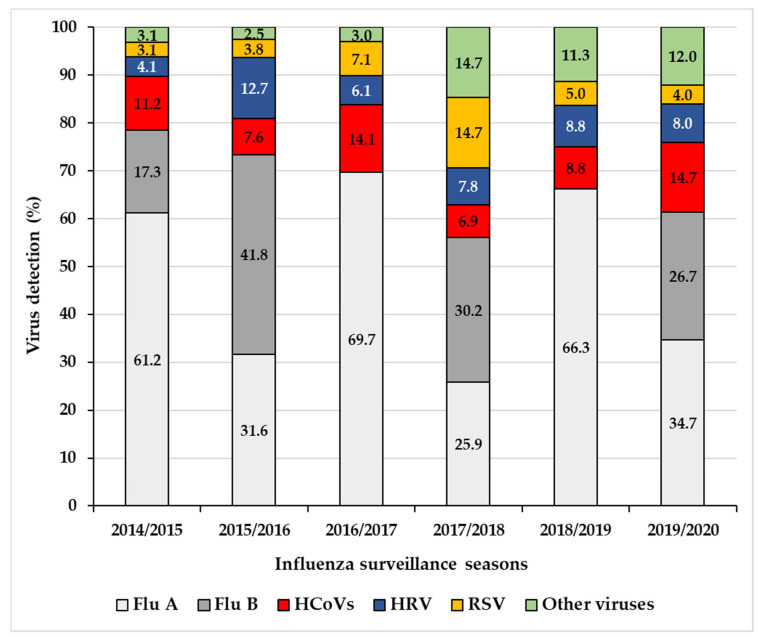
Percentages of viral respiratory infections during each influenza season (week 40 to15) from 2014/2015 to 2019/2020, based on 811 ILI/ARI patients enrolled by the general practitioners of the Corsican *Sentinelles* Network.

During the six-year study, among the 811 ILI/ARI patients enrolled by the Corsican *Sentinelles* Network, 63.3% (*n* = 513) were positive for at least one of the respiratory viruses analysed (Table 2). HCoVs were the third most frequently detected viruses (7.0%; *n* = 57) after influenza viruses subtypes A (32.4%; *n* = 263) and B (12.9%; *n* = 105) (Table 2). The number of HCoV infections ranged from 4.5% in 2014/2015 (6 of 133) to 10.0% in 2016/2017 (14 of 140) (Table 2). The contribution of HCoVs to the total number of viral detections (*n* = 547) (Figure 2) in the 811 ILI/ARI patients during each influenza season (week 40 to week 15) ranged from 6.9% (8 of 116) in 2017/2018 to 14.7% (11 of 75) in 2019/2020.

### 3.2. Seasonality of HCoVs

**Figure 3:**
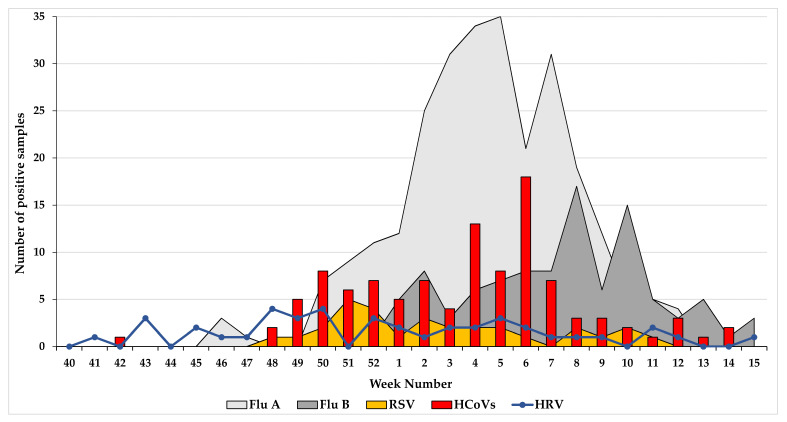
Analysis of the weekly distribution of Human Coronaviruses (HCoVs), influenza viruses (A and B), RSV, and HRV. Flu A: Influenza virus A; Flu B: Influenza virus B; HCoVs: Human coronaviruses; HRV: Human rhinovirus; RSV: Respiratory syncytial virus.

The seasonal distribution per week of HCoV, influenza viruses (A and B), RSV, and HRV was studied in 1389 ILI/ARI patients (Table 1 and Figure 3). Among the 1389 patients, 59.6% (*n* = 828) were positive for at least one respiratory virus, of which 39.6% (*n* = 328) were positive for influenza A virus, 25.6% (*n* = 212) for influenza B virus, and 12.6% (*n* = 105) for HCoVs. HCoVs were detected during the entire winter season (weeks 48–14), with the highest number of HCoVs detected in week 6 (Figure 3). Notably, week 6 had a decrease in influenza A virus detection (Figure 3). Since the number of HCoVs detected by species per season was low, the seasonality of HCoVs was established from all viruses detected per week over the six influenza seasons. The HCoV peak trend detection among week 4 to 7 was clearly observed during three influenza seasons (2014/2015; 2015/2016; 2019/2020).

**Figure 4:**
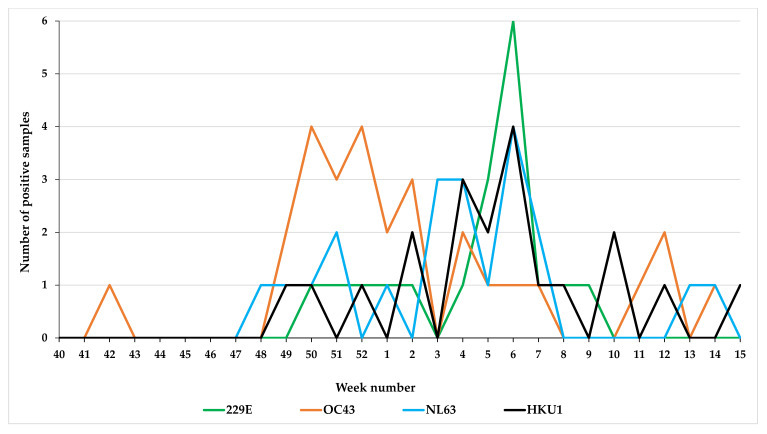
Weekly prevalence of seasonal human coronaviruses (HCoVs) detected among ILI/ARI patients.

Among the 105 HCoV cases, 82.8% (*n* = 87) were typed. Of these 32.2% (*n* = 28) of cases were infected with HCoV-OC43, 24.1% (*n* = 21) with HCoV-NL63, 22.9% (*n* = 20) with HCoV-HKU1, and 20.6% (*n* = 18) with HCoV-229E (Table 1). The weekly number of HCoV types detected among the 1389 ILI/ARI patients is illustrated in Figure 3. A degree of synchrony was observed during all winter seasons and in the timing of the peak (week 6) for HCoV-HKU1, NL63, and 229E, whereas HCoV-OC43 infections occurred earlier with a peak in weeks 50–52 (Figure 4).

### 3.3. Co-Infection in HCoV-Positive Patients

Among the 1389 ILI/ARI patients, 105 (7.5%) were positive for at least one HCoV. Among them, 82 (78.1%) were infected with one HCoV, while 23 (21.9%) were infected with at least two different respiratory viruses (including two co-infections with three viruses) (Table 3). Of these, the most frequently observed co-pathogens were influenza viruses (flu A: *n* = 9/23; 39.1%, and flu B: *n* = 8/23; 34.8%) (Table 3). Two co-infection combinations were detected: HCoV-229E/NL63 and HCoV-HKU1/OC43.

### 3.4. Age Distribution of HCoV-Positive Patients

**Figure 5:**
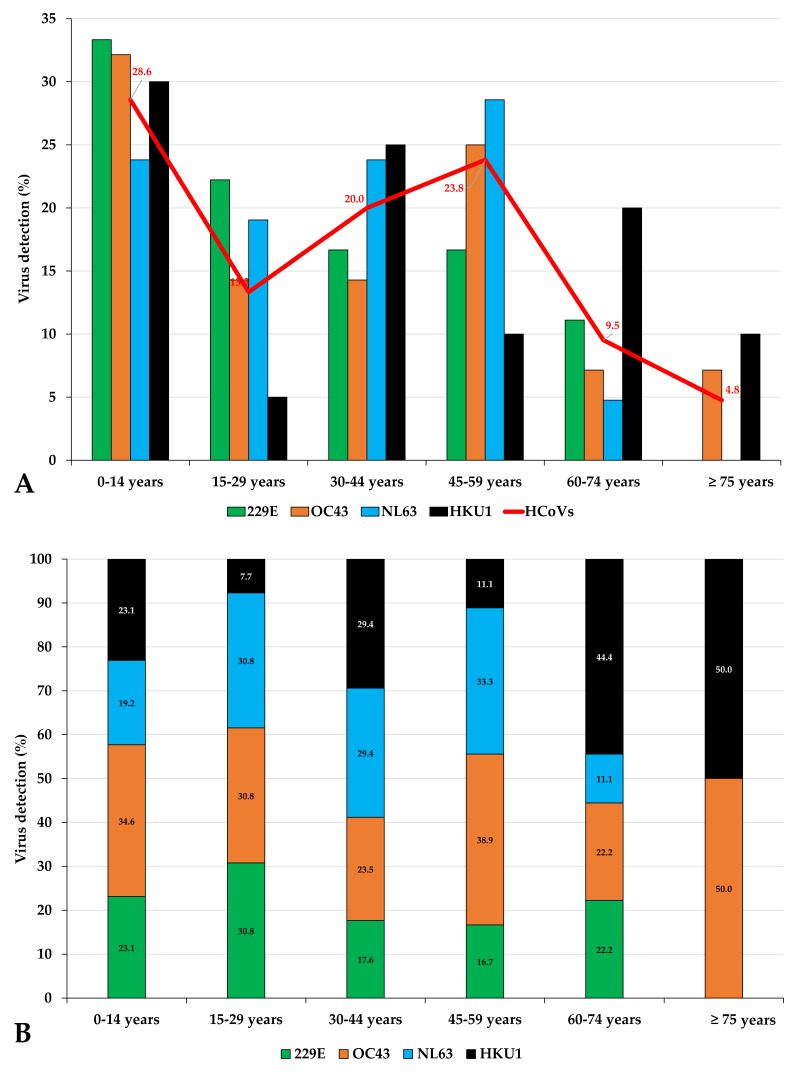
Age distribution of patients with human coronavirus infection (**A**) and the distribution of coronavirus subtype infections in the different age groups (**B**).

The demographic data and clinical characteristics of the 105 confirmed HCoV-positive patients are summarized in Table 4. The age of patients infected with HCoV varied from 1 month to 90 years with mean and median ages of 34 years. The male to female ratio of HCoV-infected patients was 1.06 (54/51). HCoV infections were observed in all age groups (Table 4 and Figure 5). However, the detection rates in these different age groups varied significantly (*p* = 0.00005) with the age group of 0–14 years accounting for 28.6% (*n* = 30) of HCoV-positive patients. In the remaining groups, detection rates were observed of 13.3% (*n* = 14) in the 15–29 age group, 20% (*n* = 21) in the 30–44 age group, 23.8% (*n* = 25) in the 45–59 age group, 9.5% (*n* = 10) in the 60–74 age group, and 4.8% (*n* = 5) in the group aged ≥75 years old.

HCoV-OC43 and HCoV-HKU1 were detected in patients of all ages (Table 5 and Figure 5). The oldest patients (≥75 years old) were infected by HCoV-OC43 and HCoV-HKU1 only (Figure 5A), and HCoV-HKU1 was the most prevalent among patients aged ≥60 years old (Figure 5B), but a significant difference in the age-group distribution of HCoV species was not observed (Table 5).

### 3.5. Clinical Characteristics of ARI/ILI Patients and Comparison of Patients according to Their Viral Infection

The epidemiological and clinical features of patients were compared according to their viral infection: influenza A cases, influenza B cases, and HCoV cases (single or co-infection). Fever and malaise were less frequent in HCoV patients than in influenza patients, while sore throat, dyspnoea, rhinorrhoea, and conjunctivitis were more frequently associated with HCoV positivity (Table 4). Sore throat and dyspnoea were more frequently detected in patients with HCoV single infection (75.3%, 55/82) than in patients with HCoV co-infection (42.9%, 9/23; *p* = 0.006) (Table 4). Notably, oseltamivir was prescribed more frequently to influenza A patients (Table 4).

Lastly, when comparing epidemiological and clinical features of patients according to the HCoV strain detected, there was a significant effect on the prevalence of sex (*p* = 0.002), with the HCoV-229E strain detected more frequently in male patients (88.9%) (Table 5). A significant difference in the prevalence of malaise (*p* = 0.025) among HCoV strain patients was also identified, as this symptom was exclusively related to HCoV-HKU1 patients (15.8%, 3/20) (Table 5).

### 3.6. Clinical Description of SARS-CoV-2-Positive Patients Seen in General Practice

Among the 119 ARI patient samples collected during the 2019/2020 influenza season and tested retrospectively for SARS-CoV-2 from week 40 to week 15, four (3.3%) were positive for SARS-CoV-2. These four infections were detected during weeks 11 (*n* = 1), 14 (*n* = 2), and 15 (*n* = 1). Fever, cough, and headache were the most commonly declared symptoms, while anosmia was reported for one patient. The mean age of the four patients was 60.75 years and the sex ratio was 1:1 (two women and two men).

## 4. Discussion

Data from continuous surveillance are very important for identifying the pattern of HCoV epidemiology. This report is the first to describe patterns of circulation of the four common HCoV strains in French ILI/ARI patients seen in general practice over six influenza seasons. HCoV was the third most detected respiratory virus in ARI/ILI patients after the influenza A and B viruses. Similar to their susceptibility to other respiratory viruses, young children aged <14 years old had the highest risk of HCoV infection. Sore throat, dyspnoea, and conjunctivitis were more frequently described among HCoV cases than among influenza cases.

Similar to other countries in the northern hemisphere, we reported a winter seasonal prevalence pattern for the four HCoV strains [23], with the highest number of cases presenting in February [9,24]. In our study, the peak in February was attributable to the 229E, NL63, and HKU1 strains, whereas the OC43 strain had peaks in December. Overall, among the HCoV-positive patients in this study, the OC43 strain was the most commonly detected virus, followed by the NL63, HKU1, and 229E strains, in agreement with previous studies [9,25,26]. HCoV-OC43 is routinely associated with ~5% of acute respiratory infections and was the most commonly detected virus in HCoV patients [27,28,29]. A seroconversion study in hospitalized children reported that HCoV-OC43 and HCoV-NL63 might induce cross-immunity, and therefore reduce the subsequent number of clinically identified HCoV-HKU1 and HCoV-229E infections [30].

We confirmed that, similar to other respiratory pathogens, HCoVs were widespread in all age groups [31]. A significant difference in the age distribution of HCoV patients was noted, with the youngest (0–14 years old) patients displaying a threefold higher level of infection than those aged 60–74 years old and a sevenfold higher infection level than patients aged ≥75 years old. Among HCoV patients aged <60 years old, the most frequently detected viruses were HCoV-OC43 and HCoV-NL63, while among patients aged ≥60 years old, HCoV-HKU1 was the most frequently detected. This trend of HCoVs infection has already been reported in other studies [14,25,26,29]. A recent surveillance study on the occurrence and hospitalization rates in children with respiratory infections over 9 years reported that HCoVs were involved in 9.1% of episodes [25]. To date, in contrast to that observed for HCoVs, few SARS-CoV-2 have been observed in children. Official national statistics in Italy reported that 1% of total cases diagnosed country-wide were below 20 years [32]. It could be possible that pre-existing cross-immunity provides protection and/or reduces the severity of COVID-19, thereby reducing the number of children who are tested/hospitalized [33]. The fewer number of HCoV infections in older than younger patients may result from higher antibody levels or other immune mechanisms of protection [34]. The lower number of HCoV infections in older than younger patients may result from higher antibody levels or other immune mechanisms of protection [34]. It is well known that HCoVs co-circulate endemically with other common respiratory viruses and co-infections are frequently observed. In our study, six other common respiratory viruses were detected simultaneously and 21.9% of the HCoV-positive patients were co-infected by at least one of the other respiratory viruses. The occurrence of co-infection of HCoV, including other HCoVs, RSV, influenza A and B viruses, HAdV, and HMPV has been reported [29,35]. Similar to other studies, we observed that influenza and RSV were the most common respiratory viruses co-infected with HCoV [27,36]. We did not observe co-infection of SARS-CoV-2 and another respiratory virus among the four COVID-19 patients detected. A recent co-infection of SARS-CoV-2 and HCoV-HKU1 has been reported [37].

In this study, we compared the demographic data and clinical characteristics of HCoV patients with those of influenza A and B patients, respectively (Table 4). Sore throat, dyspnoea, rhinorrhoea, and conjunctivitis were more often observed in HCoV cases than in influenza cases. While dyspnoea was reported as not common in seasonal influenza viruses [38], studies of hospitalized patients reported the role of HCoV in causing lower respiratory illness [13,26]. Fever was more often observed in influenza cases, as reported [23]. These observations may be of importance, especially when HCoVs and influenza virus co-circulate. The clinical impact of HCoVs in co-detection is not fully understood, with previous studies observing unchanged morbidity or increased illness severity [39]. When we compared the clinical presentations of patients with a single HCoV infection with those with co-infection, sore throat and dyspnoea were more commonly reported in cases with a single infection.

A growing number of studies are currently describing the clinical features due to SARS-CoV-2 infection [40,41,42,43,44]. Although most of the COVID-19 studies have been conducted on hospitalized patients some comparisons with data of the present study might be useful. Fever and cough were the most common symptoms reported for SARS-CoV-2 and HCoV patients [45]. Compared to symptoms observed in HCoV patients included in the present study, SARS-CoV-2 patients seem to show less headache (6.3%–33.9% vs. 80.8%), rhinorrhea (4% vs. 87.7%) and myalgia (11.1–34.8% vs. 71.9%) [45]. Sore throat, which was one of the most detected symptoms among HCoVs patients, was less common among COVID-19 patients [43]. About half of COVID-19 patients had an underlying disease [45], whereas in our study less than a quarter of the HCoV patients suffered from an underlying disease.

This study has some limitations. First, we used two ILI/ARI patient samples, which can introduce bias, even if the methodology of enrolment was very similar. Second, the number of HCoV patients included did not allow the identification of meaningful associations by subanalyses. Third, we studied HCoV circulation during influenza season and not on annual basis. As previously described, HCoVs co-circulate and showed marked winter seasonality, and are not detected in the summer period [14]. Lastly, we did not identify the species of 20 HCoV cases because relevant samples were not available.

In conclusion, this study demonstrates that HCoV subtypes appear in ARI/ILI patients seen in general practice, with characteristic outbreak patterns primarily in winter. HCoVs were detected at a significantly higher rate in children aged <14 years old than in older patients aged ≥60 years. Some clinical manifestations seem to differentiate HCoV patients from influenza patients. Further studies with representative samples should be conducted to provide additional insights into the epidemiology and clinical features of HCoVs.

## Figures and Tables

**Table 1:** Samples from 2014/2015 to 2019/2020 included in the study.

Origin of Samples	Period (Week 40 to 15)	*N*	HCoVs	229E	OC43	NL63	HKU1	Not Typed
			*n* (%)	*n* (%)	*n* (%)	*n* (%)	*n* (%)	*n* (%)
Influenza Surveillance	2014/2015 to 2019/2020	811	57 (7.0)	12 (21.0)	20 (35.1)	11 (19.3)	11 (19.3)	3 (5.3)
IRIIS	2015/2016 to 2016/2017	578	48 (8.3)	6 (12.0)	8 (16.0)	10 (20.0)	9 (18.0)	17 (34.0)
Total	2014/2015 to 2019/2020	1389	105 * (7.5)	18 (17.1)	28 (26.6)	21 (20.0)	20 (20.0)	20 (18.1)

* Among the HCoV-positive patients, two were co-infected by two HCoVs (number of HCoVs detected = 107).

**Table 2:** Chronological distribution of the different viruses detected during the influenza surveillance seasons.

	Overall	Flu A		Flu B		HCoVs		HRV		RSV		Other Viruses	
	*N*	*n*	%	*n*	%	*n*	%	*n*	%	*n*	%	*n*	%
2014/2015	151	60	39.7	17	11.3	11	7.3	4	2.6	3	2.0	3	2.0
2015/2016	133	25	18.8	33	24.8	6	4.5	10	7.5	3	2.3	2	1.5
2016/2017	140	69	49.3	0	0.0	14	10.0	6	4.3	7	5.0	3	2.1
2017/2018	150	30	20.0	35	23.3	8	5.3	9	6.0	17	11.3	17	11.3
2018/2019	118	53	44.9	0	0.0	7	5.9	7	5.9	4	3.4	9	7.6
2019/2020	119	26	21.8	20	16.8	11	9.2	6	5.0	3	2.5	9	7.6
TOTAL	811	263	32.4	105	12.9	57	7.0	42	5.2	37	4.6	43	5.3

Flu A: Influenza virus A; Flu B: Influenza virus B; HCoVs: Human coronaviruses; HRV: Human rhinovirus; RSV: Respiratory syncytial virus.

**Table 3:** Co-pathogen detection in human coronavirus-positive patients.

Co-Pathogen	HCoVs	229E	OC43	NL63	HKU1	HCoV Without Species Determination
Flu A	9	5	1	/	2	1
Flu B	8	/	1	2	3	2
RSV	3	/	/	1	2	/
HRV	1	1	/	/	/	/
HMPV	1	/	/	/	1	/
HAdV	1	/	/	1	/	/
HCoV-229E			/	1	/	/
HCoV-OC43		/		/	1	/
HCoV-NL63		1	/		/	/
HCoV-HKU1		/	1	/		/
HCoV without species determination		/	/	/	/	
Total	23 *	7	3	5	9	3

* Two co-infections with three viruses (A(H1N1)pdm09/HKU/HMPV and BYAM/NL63/HAdV) and two co-infections with two HCoVs (HCoV-229E/NL63 and HCoV-HKU1/OC43). Flu A: Influenza virus A; Flu B: Influenza virus B; HCoVs: Human coronaviruses; HRV: Human rhinovirus; RSV: Respiratory syncytial virus; HMPV: Human metapneumovirus; HAdV: Human adenovirus.

**Table 4:** Age, sex and clinical characteristics of ARI/ILI patients and their positivity for human coronaviruses and influenza viruses.

**Collected Variable**		**All Patients ARI./ILI**			**Patients positive**						**Patients HCoVs Positive**													**Negative for All Viruses Tested**		
					**Influenza Virus A**			**Influenza Virus B**			All HCoVs Positive Patients*			***p*-Value**		**Single HCoV**			**Co-Pathogen**			***p*-Value**				***p*-Value**
		***n***	**%**		***n***	**%**		***n***	**%**		***n***	**%**		**FluA/HCoV**	**FluB/HCoV**	***n***	**%**		***n***	**%**		**singleHCoV/ Copathogen**	***n***	**%**		**HCoV/ Negative**
Number of HCoVs positive patients		1389	100.0		328	23.6		212	15.3		105	7.6				82	78.1		23	21.9			561	40.4		
Age	Median Age (years)	34			36			28.5			34			/	/	39.5			11.0			/		36.0		/
	Mean Age (Min-Max) (years)	34.96 (0.08–97.0)			34.53 (0.08–89)			31.02 (0.67–91)			34.61 (0.08–90)					37.43 (0.50–90.0)			24.5 (0.08–80)					36.73 (0.25–90)		
	Age IQR (years)	13–53			13–51			10–48			11–52					16.0–53.5			3.5–44.0					17.0–54.0		
		***n***	**%**		***n***	**%**		***n***	**%**		***n***	**%**	***p*-Value**			**n**	**%**	***p*-Value**	***n***	**%**	***p*-Value**		***n***	**%**		
	0–14 years	378	27.2		88	26.8		73	34.4		30	28.6	**0.00005**	0.908	0.535	18	22.0	**0.00137**	12	52.2	**0.00005**	**0.008**	124	22.1		0.406
	15–29 years	210	15.1		42	12.8		37	17.5		14	13.3				13	15.9		1	4.3		0.295	93	16.6		
	30–44 years	303	21.8		84	25.6		42	19.8		21	20.0				17	20.7		4	17.4		1	135	24.1		
	45–59 years	268	19.3		69	21.0		34	16.0		25	23.8				22	26.8		3	13.0		0.267	104	18.5		
	60–74 years	149	10.7		32	9.8		16	7.5		10	9.5				8	9.8		2	8.7		1	73	13.0		
	≥ 75	81	5.8		13	4.0		10	4.7		5	4.8				4	4.9		1	4.3		1	32	5.7		
Sex	Female	758	54.6		184	56.1		110	51.9		51	48.6	0.782	0.216	0.633	44	53.7	0.435	7	30.4	**0.0174**	0.06	311	55.4		0.202
	Male	631	45.4		144	43.9		102	48.1		54	51.4				38	46.3		16	69.6			250	44.6		
		***n***	**%**	**NA**	***n***	**%**	**NA**	***n***	**%**	**NA**	***n***	**%**	**NA**			***n***	**%**	**NA**	***n***	**%**	**NA**		***n***	**%**	**NA**	
Symptoms	Fever	1298	95.2	25	321	98.5	2	205	97.2	1	94	91.3	2	**0.00131**	**0.0439**	72	88.9	1	22	100.0	1	0.198	513	93.4	12	0.400
	Cough	1087	87.8	151	247	92.2	60	173	88.7	17	84	89.4	11	0.397	1	66	90.4	9	18	85.7	2	0.688	418	82.9	57	0.128
	Sore throat	659	53.2	151	125	46.6	60	101	51.8	17	64	68.1	11	**0.000465**	**0.011**	55	75.3	9	9	42.9	2	**0.006**	267	53.0	57	**0.0068**
	Dyspnea	241	19.5	151	49	14.9	0	31	14.6	0	32	30.5	0	**0.000817**	**0.00152**	29	35.4	0	3	13.0	0	**0.04**	91	16.2	0	**0.000963**
	Rhinorrhea	932	75.3	151	206	76.9	60	150	76.9	17	83	88.3	11	**0.017**	**0.0257**	64	87.7	9	19	90.5	2	1	355	70.4	57	**0.000201**
	Myalgia	666	69.0	424	123	70.7	154	120	74.1	50	52	70.3	31	1	0.533	41	71.9	25	11	64.7	6	0.561	288	71.5	158	0.889
	Headache	863	69.7	151	194	72.4	60	147	75.4	17	73	77.7	11	0.343	0.769	59	80.8	9	14	66.7	2	0.233	346	68.7	57	0.0866
	Malaise	85	12.0	681	26	12.7	124	13	14.3	121	3	3.2	11	**0.0104**	**0.00844**	2	2.7	9	1	4.8	2	0.536	36	14.9	319	0.002
	Conjunctivitis	345	24.8	0	67	20.4	0	59	27.8	0	32	30.5	0	**0.0446**	0.692	24	29.3	0	8	34.8	0	0.616	141	25.1	0	0.275
	Vomiting	151	10.9	0	32	9.8	0	20	9.4	0	15	14.3	0	0.208	0.252	12	14.6	0	3	13.0	0	1	66	11.8	0	0.514
	Diarrhea	154	11.1	0	23	7.0	0	23	10.8	0	10	9.5	0	0.401	0.845	9	11.0	0	1	4.3	0	0.442	68	12.1	0	0.512
	Other symptoms	184	21.0	512	44	16.2	56	21	18.3	97	18	26.9	38	0.0521	0.192	15	30.6	33	3	16.7	5	0.757	82	25.0	233	0.759
	Anormal lung auscultation	88	26.3	1055	24	25.3	233	8	17.0	165	8	38.1	84	0.282	0.0709	6	46.2	69	2	25.0	15	0.4	34	27.2	436	0.309
Other variables	Risk factors	321	23.5	22	59	18.3	6	41	19.4	1	16	15.2	0	0.555	0.4	13	15.9	0	3	13.0	0	1	145	26.4	11	**0.0182**
	Oseltamivir	241	18.0	49	88	27.7	10	29	14.0	5	14	13.6	2	**0.00348**	1	12	15.0	2	2	8.7	0	0.73	88	16.4	26	0.558
	Antibiotics	173	13.3	86	43	14.2	25	18	8.8	7	10	9.9	4	0.31	0.833	7	8.9	3	3	13.6	1	0.45	73	14.1	43	0.338
	Hospitalization	39	2.9	32	12	3.8	10	7	3.4	5	2	1.9	0	0.532	0.7	2	2.4	0	0	0.0	0	1	15	2.7	10	1

* Overall HCoV infections also include strains without species determination (*N* = 20).

**Table 5:** Description of HCoV-positive patients according to the viral strains detected.

**Collected Variable**		**Distribution of HCoV Strain**												
		**229E**			**OC43**			**NL63**			**HKU1**			***p*-Value Variation between Species**
		***n***	**%**		***n***	**%**		***n***	**%**		***n***	**%**		
Number of HCoVs positive patients		18	17.1		28	26.7		21	20.0		20	19.0		
Age	Median Age (years)	27			31			40			34.5			/
	Mean Age (Min-Max) (years)	31.1 (4–69)			33.1 (0.5–90.0)			33.72 (0.08–68)			36.99 (0.83–80)			
	Age IQR (years)	11.25–47.5			7–48.25			15–52			4–60.25			
		***n***	**%**	***p*-Value**	***n***	**%**	***p*-Value**	***n***	**%**	***p*-Value**	***n***	**%**	***p*-Value**	
	0–14 years	6	33.3	0.156	9	32.1	0.0721	5	23.8	0.0721	6	30.0	0.224	0.927
	15–29 years	4	22.2		4	14.3		4	19.0		1	5.0		0.46
	30–44 years	3	16.7		4	14.3		5	23.8		5	25.0		0.75
	45–59 years	3	16.7		7	25.0		6	28.6		2	10.0		0.44
	60–74 years	2	11.1		2	7.1		1	4.8		4	20.0		0.38
	≥ 75	0	0.0		2	7.1		0	0.0		2	10.0		0.3
Sex	Female	2	11.1	0.0000005	15	53.6	0.79	13	61.9	0.217	11	55.0	0.752	**0.00209**
	Male	16	88.9		13	46.4		8	38.1		9	45.0		
		***n***	**%**	**NA**	***n***	**%**	**NA**	***n***	**%**	**NA**	***n***	**%**	**NA**	
Symptoms	Fever	17	94.4	0	26	92.9	0	16	84.2	2	19	95.0	0	0.140
	Cough	14	100.0	4	22	91.7	4	17	89.5	2	15	78.9	1	0.261
	Sore throat	11	78.6	4	14	58.3	4	15	78.9	2	11	57.9	1	0.307
	Dyspnea	3	16.7	0	4	14.3	0	7	33.3	0	8	40.0	0	0.138
	Rhinorrhea	13	92.9	4	23	95.8	4	16	84.2	2	16	84.2	1	0.509
	Myalgia	7	77.8	9	10	66.7	13	13	76.5	4	12	66.7	2	0.861
	Headache	13	92.9	4	20	83.3	4	14	73.7	2	13	68.4	1	0.322
	Malaise	0	0.0	4	0	0.0	4	0	0.0	2	3	15.8	1	**0.0247**
	Conjunctivitis	4	22.2	0	11	39.3	0	6	28.6	0	6	30.0	0	0.656
	Vomiting	1	5.6	0	5	17.9	0	4	19.0	0	2	10.0	0	0.543
	Diarrhea	1	5.6	0	1	3.6	0	2	9.5	0	3	15.0	0	0.517
	Other symptoms	4	30.8	5	3	15.0	8	2	15.4	8	3	25.0	8	0.669
	Anormal lung auscultation	1	33.3	15	4	66.7	22	1	16.7	15	2	33.3	14	0.343
Other variables	Risk factors	0	0.0	0	3	10.7	0	3	14.3	0	5	25.0	0	0.138
	Oseltamivir	3	16.7	0	2	7.1	0	3	15.8	2	4	20.0	0	0.607
	Antibiotics	2	11.1	0	1	3.6	0	3	15.0	1	1	5.9	3	0.515
	Hospitalization	1	5.6	0	0	0.0	0	0	0.0	0	0	0.0	0	0.275

## References

[1] Cui J., Li F., Shi Z. (2018). Origin and evolution of pathogenic coronaviruses. Nat. Rev. Genet..

[2] Cabeça T.K., Granato C., Bellei N.C.J. (2013). Epidemiological and clinical features of human coronavirus infections among different subsets of patients. Influ. Other Respir. Viruses.

[3] Esper F., Ou Z., Huang Y.T. (2010). Human coronaviruses are uncommon in patients with gastrointestinal illness. J. Clin. Virol..

[4] Perlman S., Netland J. (2009). Coronaviruses post-SARS: Update on replication and pathogenesis. Nat. Rev. Genet..

[5] WHO (2020). SARS (Severe Acute Respiratory Syndrome).

[6] Zaki A.M., Van Boheemen S., Bestebroer T., Osterhaus A., Fouchier R. (2012). Isolation of a Novel Coronavirus from a Man with Pneumonia in Saudi Arabia. N. Engl. J. Med..

[7] WHO (2020). Coronavirus Disease (COVID-19) Outbreak.

[8] Graham N.M.H. (1990). THE EPIDEMIOLOGY OF ACUTE RESPIRATORY INFECTIONS IN CHILDREN AND ADULTS: A GLOBAL PERSPECTIVE. Epidemiol. Rev..

[9] Friedman N., Alter H., Hindiyeh M., Mendelson E., Shemer-Avni Y., Mandelboim M. (2018). Human Coronavirus Infections in Israel: Epidemiology, Clinical Symptoms and Summer Seasonality of HCoV-HKU1. Viruses.

[10] Hamre D., Procknow J.J. (1966). A New Virus Isolated from the Human Respiratory Tract?. Exp. Boil. Med..

[11] Almeida J.D., Tyrrell D.A.J. (1967). The Morphology of Three Previously Uncharacterized Human Respiratory Viruses that Grow in Organ Culture. J. Gen. Virol..

[12] Van Der Hoek L., Pyrc K., Jebbink M.F., Vermeulen-Oost W., Berkhout R.J.M., Wolthers K.C., Dillen P.M.E.W.-V., Kaandorp J., Spaargaren J., Berkhout B. (2004). Identification of a new human coronavirus. Nat. Med..

[13] Woo P.C.Y., Lau S.K.P., Chu C.-M., Chan K.-H., Tsoi H.-W., Huang Y., Wong B.H.L., Poon R.W.S., Cai J.J., Luk W.-K. (2005). Characterization and Complete Genome Sequence of a Novel Coronavirus, Coronavirus HKU1, from Patients with Pneumonia. J. Virol..

[14] Gaunt E.R., Hardie A., Claas E.C.J., Simmonds P., Templeton K.E. (2010). Epidemiology and Clinical Presentations of the Four Human Coronaviruses 229E, HKU1, NL63, and OC43 Detected over 3 Years Using a Novel Multiplex Real-Time PCR Method. J. Clin. Microbiol..

[15] Tang J.W., Lam T.T.-Y., Zaraket H., Lipkin W.I., Drews S.J., Hatchette T.F., Heraud J.-M., Koopmans M.P.G., Abraham A., Baraket A. (2017). Global epidemiology of non-influenza RNA respiratory viruses: Data gaps and a growing need for surveillance. Lancet Infect. Dis..

[16] Jean A., Quach C., Yung A., Semret M. (2013). Severity and Outcome Associated With Human Coronavirus OC43 Infections Among Children. Pediatr. Infect. Dis. J..

[17] Gagneur A., Vallet S., Talbot P.J., Legrand-Quillien M., Picard B., Payan C., Sizun J. (2008). Outbreaks of human coronavirus in a pediatric and neonatal intensive care unit. Eur. J. Pediatr..

[18] Hand J., Rose E.B., Salinas A., Lu X., Sakthivel S.K., Schneider E., Watson J.T. (2018). Severe Respiratory Illness Outbreak Associated with Human Coronavirus NL63 in a Long-Term Care Facility. Emerg. Infect. Dis..

[19] Kozak R., Prost K., Yip L., Williams V., Leis J.A., Mubareka S. (2020). Severity of coronavirus respiratory tract infections in adults admitted to acute care in Toronto, Ontario. J. Clin. Virol..

[20] Minodier L., Masse S., Capai L., Blanchon T., Ceccaldi P., Werf S., Hanslik T., Charrel R., Falchi A. (2019). Risk factors for seasonal influenza virus detection in stools of patients consulting in general practice for acute respiratory infections in France, 2014-2016. Influ. Other Respir. Viruses.

[21] Corman V.M., Landt O., Kaiser M., Molenkamp R., Meijer A., Chu D.K., Bleicker T., Brünink S., Schneider J., Schmidt M.L. (2020). Detection of 2019 novel coronavirus (2019-nCoV) by real-time RT-PCR. Eurosurveillance.

[22] WHO (2020). Real-Time Rt-Pcr-Assays for the Detection of SARS-CoV-2 Institut Pasteur Paris.

[23] Zimmermann P., Curtis N. (2020). Coronavirus Infections in Children Including COVID-19. Pediatr. Infect. Dis. J..

[24] Jevšnik M., Uršič T., Žigon N., Lusa L., Krivec U., Petrovec M. (2012). Coronavirus infections in hospitalized pediatric patients with acute respiratory tract disease. BMC Infect. Dis..

[25] Heimdal I., Moe N., Krokstad S., Christensen A., Skanke L.H., Nordbø S.A., Døllner H. (2019). Human Coronavirus in Hospitalized Children With Respiratory Tract Infections: A 9-Year Population-Based Study From Norway. J. Infect. Dis..

[26] Monto A.S., Dejonge P.M., Callear A.P., Bazzi L.A., Capriola S.B., Malosh R.E., Martin E.T., Petrie J.G. (2020). Coronavirus Occurrence and Transmission Over 8 Years in the HIVE Cohort of Households in Michigan. J. Infect. Dis..

[27] Zeng Z.-Q., Chen D.-H., Tan W.-P., Qiu S.-Y., Xu D., Liang H.-X., Chen M.-X., Li X., Lin Z.-S., Liu W.-K. (2017). Epidemiology and clinical characteristics of human coronaviruses OC43, 229E, NL63, and HKU1: A study of hospitalized children with acute respiratory tract infection in Guangzhou, China. Eur. J. Clin. Microbiol. Infect. Dis..

[28] Varghese L., Zachariah P., Vargas C., LaRussa P., Demmer R.T., Furuya Y.E., Whittier S., Reed C., Stockwell M.S., Saiman L. (2017). Epidemiology and Clinical Features of Human Coronaviruses in the Pediatric Population. J. Pediatr. Infect. Dis. Soc..

[29] Killerby M.E., Biggs H.M., Haynes A., Dahl R.M., Mustaquim D., Gerber S.I., Watson J.T. (2018). Human coronavirus circulation in the United States 2014–2017. J. Clin. Virol..

[30] Dijkman R., Jebbink M.F., Gaunt E.R., Rossen J.W., Templeton K.E., Kuijpers T.W., Van Der Hoek L. (2012). The dominance of human coronavirus OC43 and NL63 infections in infants. J. Clin. Virol..

[31] Vabret A., Brouard J., Petitjean J., Eugene-Ruellan G., Freymuth F. (1998). Human coronavirus infections: Importance and diagnosis. La Presse Médicale.

[32] Parri N., Magistà A.M., Marchetti F., Cantoni B., Arrighini A., Romanengo M., Felici E., Urbino A., Da Dalt L., Verdoni L. (2020). Characteristic of COVID-19 infection in pediatric patients: Early findings from two Italian Pediatric Research Networks. Eur. J. Nucl. Med. Mol. Imaging.

[33] Nickbakhsh S., Ho A., Marques D.F.P., McMenamin J., Gunson R.N., Murcia P.R. (2020). Epidemiology of Seasonal Coronaviruses: Establishing the Context for the Emergence of Coronavirus Disease 2019. J. Infect. Dis..

[34] Gorse G.J., Donovan M.M., Patel G.B. (2020). Antibodies to coronaviruses are higher in older compared with younger adults and binding antibodies are more sensitive than neutralizing antibodies in identifying coronavirus-associated illnesses. J. Med. Virol..

[35] Yu X., Lu R., Wang Z., Zhu N., Wang W., Julian D., Chris B., Lu J., Tan W. (2012). Etiology and clinical characterization of respiratory virus infections in adult patients attending an emergency department in Beijing. PLoS ONE.

[36] Debiaggi M., Canducci F., Ceresola E.R., Clementi M. (2012). The role of infections and coinfections with newly identified and emerging respiratory viruses in children. Virol. J..

[37] Chaung J., Chan D., Pada S., Tambyah P.A. (2020). Coinfection with COVID-19 and Coronavirus HKU1—The critical need for repeat testing if clinically indicated. J. Med. Virol..

[38] Jiang C., Yao X., Zhao Y., Wu J., Huang P., Pan C., Liu S., Pan C. (2020). Comparative review of respiratory diseases caused by coronaviruses and influenza A viruses during epidemic season. Microbes Infect..

[39] Asner S.A., Michelle E., Tran D., Smieja M., Merglen A., Mertz D. (2014). Clinical Disease Severity of Respiratory Viral Co-Infection versus Single Viral Infection: A Systematic Review and Meta-Analysis. PLoS ONE.

[40] Huang C., Wang Y., Li X., Ren L., Zhao J., Hu Y., Zhang L., Fan G., Xu J., Gu X. (2020). Clinical features of patients infected with 2019 novel coronavirus in Wuhan, China. Lancet.

[41] Chen N., Zhou M., Dong X., Qu J., Gong F., Han Y., Qiu Y., Wang J., Liu Y., Wei Y. (2020). Epidemiological and clinical characteristics of 99 cases of 2019 novel coronavirus pneumonia in Wuhan, China: A descriptive study. Lancet.

[42] Wang D., Hu B., Hu C., Zhu F., Liu X., Zhang J., Wang B., Xiang H., Cheng Z., Xiong Y. (2020). Clinical Characteristics of 138 Hospitalized Patients With 2019 Novel Coronavirus–Infected Pneumonia in Wuhan, China. JAMA.

[43] Xu X.-W., Wu X.-X., Jiang X.-G., Xu K.-J., Ying L.-J., Ma C.-L., Li S.-B., Wang H.-Y., Zhang S., Gao H.-N. (2020). Clinical findings in a group of patients infected with the 2019 novel coronavirus (SARS-Cov-2) outside of Wuhan, China: Retrospective case series. BMJ.

[44] Guan W.-J., Ni Z.-Y., Hu Y., Liang W.-H., Ou C.-Q., He J.-X., Liu L., Shan H., Lei C.-L., Hui D.S. (2020). Clinical Characteristics of Coronavirus Disease 2019 in China. N. Engl. J. Med..

[45] Ge H., Wang X., Yuan X., Xiao G., Wang C., Deng T., Yuan Q., Xiao X. (2020). The epidemiology and clinical information about COVID-19. Eur. J. Clin. Microbiol. Infect. Dis..

